# Acute Cardiomyopathy and Delayed Neuropsychiatric Sequelae of Carbon Monoxide Poisoning: A Case Report

**DOI:** 10.7759/cureus.42701

**Published:** 2023-07-30

**Authors:** Khaled Mohamed S Alarbi, Mohamed A Baghi, Irfan Varikkodan, Jaseem Sirajudeen, Fuad Al-Aani, Nishan Purayil, Vamanjore A Naushad

**Affiliations:** 1 Internal Medicine, Hamad Medical Corporation, Doha, QAT; 2 Internal Medicine, Hamad General Hospital, Doha, QAT; 3 Clinical Medicine, Weill Cornell Medicine - Qatar, Doha, QAT; 4 Clinical Department, College of Medicine, Qatar University, Doha, QAT; 5 Internal Medicine, Hamad Medical corporation, Doha, QAT

**Keywords:** toxicity, co, neuropsychiatry, cardiomyopathy, carbon monoxide poisoning

## Abstract

Carbon monoxide (CO) is an odorless and colorless gas that is formed by the combustion of hydrocarbon. CO poisoning is not an uncommon phenomenon that can have serious consequences for morbidity and mortality. The most frequent causes of accidental poisoning include smoke inhalation from fires, malfunctioning heating devices, smoke from motor vehicles in a poorly ventilated or enclosed space, and electrical cable fires. CO has a higher affinity to hemoglobin than oxygen leading to the formation of carboxyhemoglobin. It impairs the oxygen transport and cytochrome chain which, therefore, leads to further cellular and immunological changes. Here, we present a case of CO poisoning resulting in combined cardiac and neuropsychiatric complications.

## Introduction

Carbon monoxide (CO) is an odorless, colorless, tasteless, and non-irritating toxic gas, which is primarily produced due to the incomplete combustion of organic compounds. CO poisoning is estimated to occur in 50000 people annually in the United States and the vast majority are non-fire-related smoke inhalation with annual deaths incidence between 1000 and 1300 people per year [1].

CO avidly binds to hemoglobin, which has an affinity for CO that is 200 times greater than that of oxygen, resulting in the formation of carboxyhemoglobin. This induces an allosteric change that significantly diminishes the capability of the hemoglobin to release oxygen to peripheral tissues [2]. The clinical manifestations of CO poisoning are extremely variable and tend to correlate well with the peak blood carboxyhemoglobin levels from mild non-specific symptoms like headache, dizziness, nausea, and myalgia to more severe symptoms such as acute myocardial injury, ventricular arrhythmias, seizures, coma, or even death. Moreover, severe CO toxicity is associated with long-term neurocognitive deficits and increased long-term mortality [3]. Even though CO poisoning is not an uncommon phenomenon, severe toxicity involving the cardiovascular and central nervous system in the same patient is rare. Here, we report a case of CO poisoning complicated with acute cardiomyopathy that fully recovered within a week. One month later, the patient was admitted to the hospital with delayed neuropsychiatric sequelae.

## Case presentation

 A 38-year-old Asian woman with no prior significant illness was brought to the Emergency Department after being found in an unconscious state in her apartment. According to the patient's roommate, they were exposed to smoke from charcoal burning in their room, and she then lost consciousness. There was no history suggestive of convulsions. On arrival at the Emergency Department, her vital signs were as follows: pulse rate: 130/minute; blood pressure: 115/90mmHg; respiratory rate: 20/minute; oxygen saturation: 100% on 15 liters supplemental oxygen; and oral temperature: 36.5°C. She was in an acute confusional state with a Glasgow Coma Scale (GCS) of 11/15. The rest of the systemic examination was unremarkable. A clinical suspicion of CO poisoning was made, and the high-flow oxygen treatment was continued.

**Figure 1:**
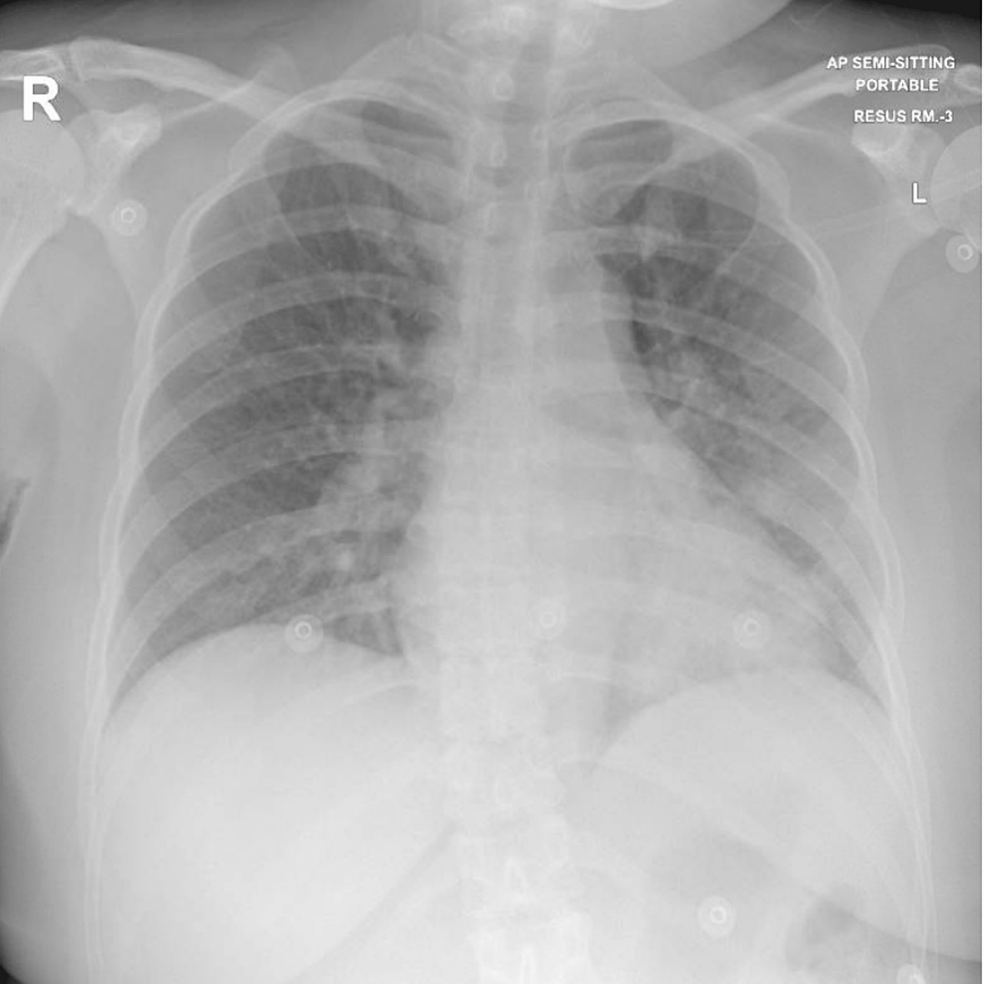
Initial chest X-ray showing cardiomegaly and non-homogenous opacities in mid and lower zones of the left lung

**Figure 2:**
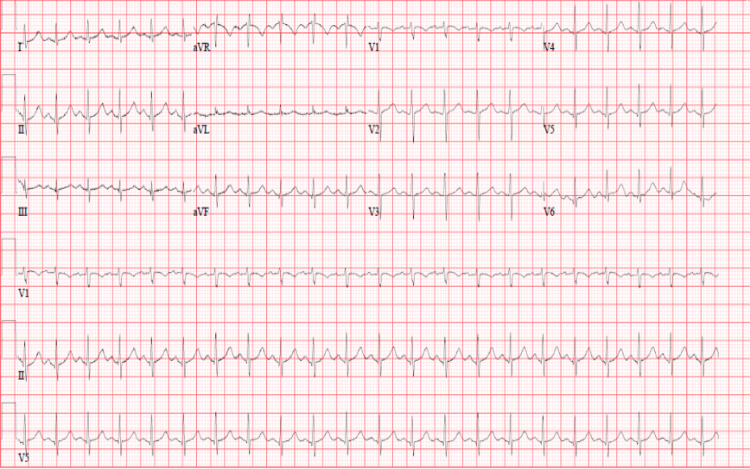
Initial ECG showing sinus tachycardia ECG, electrocardiogram

Initial laboratory results showed leukocytosis of 24.1X10^3^ cells/uL, raised high-sensitive troponin T (HsTnT) of 166 ng/L, normal renal function, and lactic acid. The carboxyhemoglobin level was elevated (11.6%). Details of the results of the initial investigations are shown in Table 1. Chest X-ray showed cardiomegaly with bilateral prominent vascular markings and nonhomogeneous opacities in the left mid and lower zones (Figure 1). The computed tomography (CT) head was normal. Electrocardiogram (ECG) showed only sinus tachycardia (Figure 2). Her clinical condition improved, and her sensorium became normal (GCS - 15/15) within 4 hours.

**Table 1:** Showing the results of laboratory investigations Pro-BNP, pro-brain natriuretic peptide; HsTnT, highly sensitive troponin T

Day	Day 1	Day 2	Day 6	Day 28	Reference range
White blood cell count (x10^3^/uL)	24.1	14.6	11.6	7.4	4-10
Hemoglobin (gm/dL)	14.5	14.7	14.5	12.6	12-15
Platelet count (x10^3^/uL)	328	274	302	246	150-410
Bicarbonate (mmol/L)	12	17	26	22	22-29
Urea (mmol/L)	6	-	8.5	3	2.5-7.8
Creatinine (umol/L)	66	56	70	59	44-80
Carboxyhemoglobin (%)	11.6	1.1	-	1	0.5-1.5
HsTnT (ng/L)	166	170	198	-	3-10
Pro-BNP (pg/mL)	-	3678	1075	-	<125

On the second day, the patient developed shortness of breath associated with orthopnea. There was no associated chest pain, cough, or fever. Physical examination revealed features of respiratory distress. Her vital signs were as follows: pulse rate: 128 beats/minute; respiratory rate: 28/minute; blood pressure: 154/94 mmHg; and oxygen saturation: 88% on room air which increased to 95% with 6 liters of oxygen. There was no cyanosis or pedal edema. A cardiovascular examination revealed raised jugular venous pressure and gallop rhythm. There were bilateral fine basal crepitations on auscultation. The rest of the systemic examination was normal.

**Figure 3:**
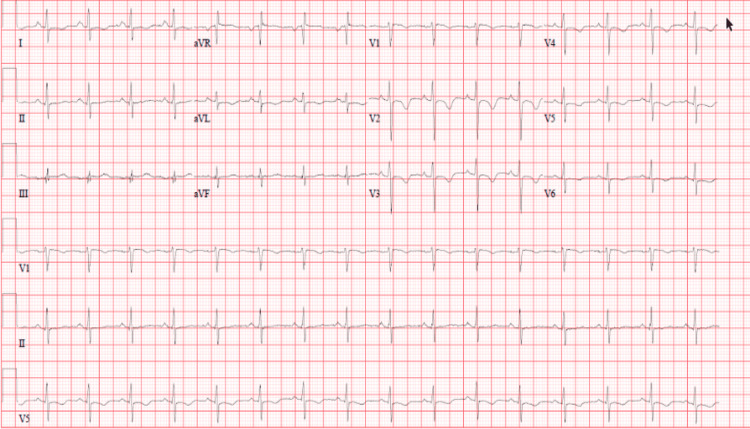
Repeat ECG showing sinus tachycardia and inverted T-waves in leads V2-V4 ECG, electrocardiogram

**Figure 4:**
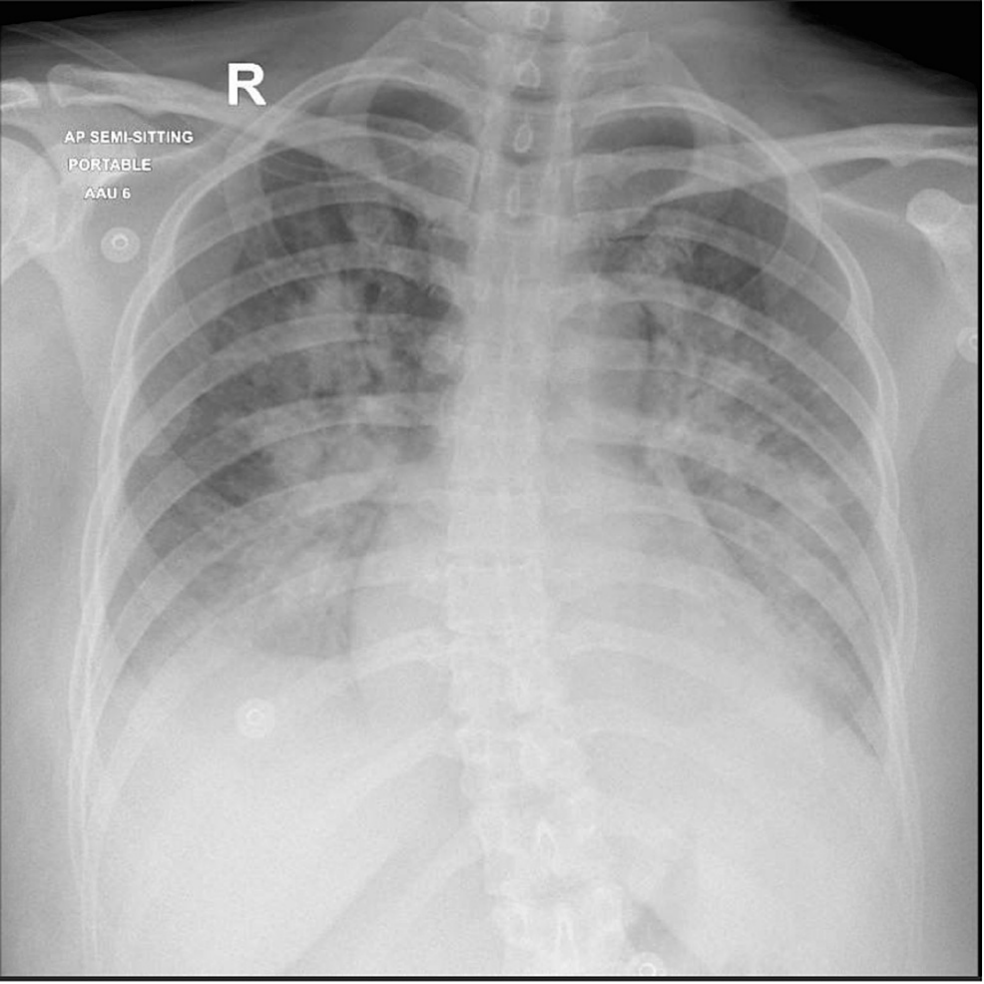
Repeat chest X-ray showing features of diffuse pulmonary edema

The repeat laboratory results showed leukocytosis but on a decreasing trend, high levels of cardiac markers, including HsTnT and natriuretic peptide test (Pro-BNP), and normalization of the carboxyhemoglobin level (Table 1). ECG and chest X-ray were repeated, which showed sinus tachycardia with inverted T-waves in V2-V4 leads (Figure 3) and diffuse pulmonary congestion (Figure 4), respectively.

**Figure 5:**
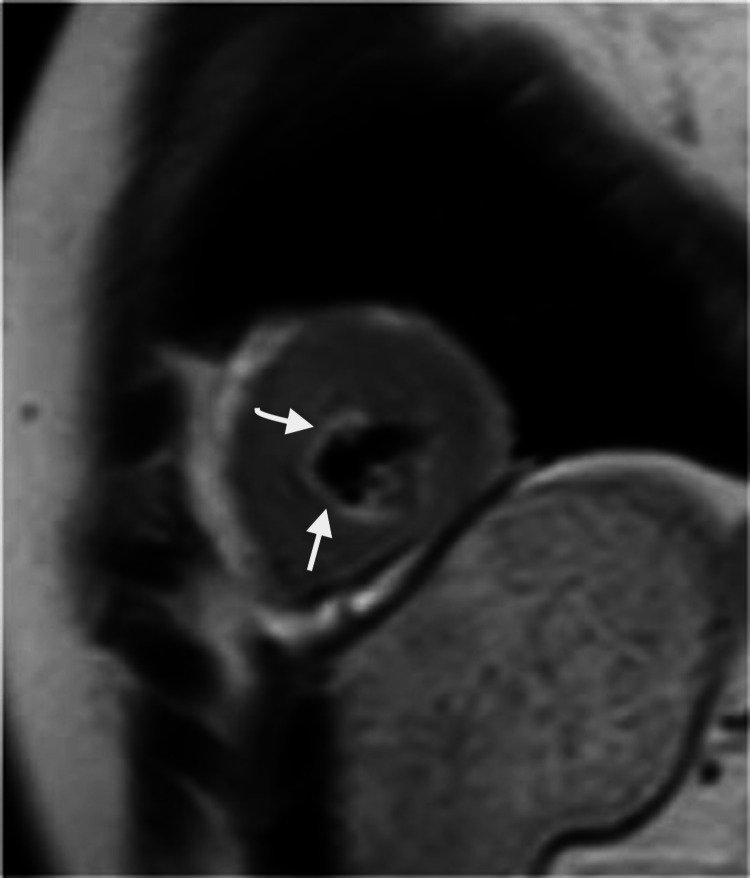
Gadolinium contrast-enhanced cardiac MRI showing subtle mid-septal to apical septal and inferior wall linear endocardial enhancement (white arrow). MRI, magnetic resonance imaging

Transthoracic echocardiography showed severely reduced left ventricular systolic function with an ejection fraction of 30%, regional wall motion abnormality, and grade 1 diastolic dysfunction. Since the patient had features of acute heart failure, a cardiology consultation was done, and she was treated with intravenous furosemide, metoprolol, and ramipril. Her symptoms improved gradually with the treatment, and her vital signs returned to normal. As a part of further cardiac evaluation, she underwent a CT coronary angiogram that was normal and a cardiac magnetic resonance imaging (MRI) that revealed features of myocarditis with mild to moderately impaired ejection fraction (43%) (Figure 5).

Repeat transthoracic echocardiogram on the sixth day prior to discharge demonstrated an improvement in the left ventricular function with an ejection fraction of 62%. She was discharged after six days of hospital stay with advice to follow up with the cardiology outpatient clinic one month later. 

The patient was readmitted to the hospital three weeks after discharge with abnormal behavior in the form of inattentiveness, frequent forgetfulness, cognitive impairment, insomnia, reduced appetite, and irrelevant speech. According to her friend, the patient has been behaving abnormally over the past four days, like removing her clothes and passing urine and stool on the bedsheet, also the patient keeps walking and wandering the whole day without rest. No harm was done to self or others. Moreover, she became dependent for her day-to-day activities such as dressing, showering, and feeding. On clinical examination, vital signs were normal. Central nervous system examination revealed severe cognitive dysfunction with apraxia. The patient's mini-mental state examination (MMSE) score was quite low, which was attributed to poor comprehension.

**Figure 6:**
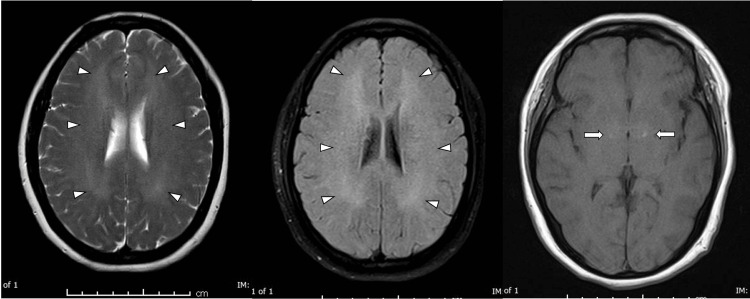
MRI of the brain showing mild diffuse T2 and FLAIR hyperintensity affecting the white matter (arrowheads). Subtle T1 hyperintensity is seen in the globus pallidus bilaterally (arrows). MRI, magnetic resonance imaging; FLAIR, fluid-attenuated inversion recovery

The blood investigations (Table 1) and CT imaging of the brain were normal. MRI of the brain showed multiple diffuse white matter lesions including external capsule, basal ganglia, thalami, bilateral globus pallidi, and mid corpus callosum and splenium (Figure 6). She was evaluated by the neurologist and mental health team. A diagnosis of delayed neuropsychiatry complications due to CO toxicity was made, and she was admitted to mental health service for further supportive care.

## Discussion

CO poisoning is a life-threatening condition that can be fatal and has been reported in both developed and developing countries [4-7]. The heart and brain are the two most common organs affected by CO poisoning. These organs have high oxygen demands, which make them prone to toxic cellular damage. The affinity of CO to hemoglobin to form carboxyhemoglobin is significantly greater than that of oxygen [8]. In addition to tissue hypoxia various other pathophysiological mechanisms have been identified as a cause for the toxic effects of CO poisoning. The mechanisms of cell injury leading to cardiac and neurological toxicity have different pathways. CO exposure induces myocardial injury by direct damage at cellular and sub-cellular levels. The effect of CO on myocardium ranges from angina, myocardial infarction, cardiomyopathy, arrhythmias, heart failure, myocardial stunning, and cardiogenic shock [9,10]. The proposed theory for such effect is summed up into the following: a) catecholamine surge, b) mitochondrial cytochrome c oxidase inhibition, and c) toxic myocarditis. Factors determining the severity and location of CO-induced cardiomyopathy are not clearly understood. Duration, concentration, dose of exposure, age, and genetic and hormonal factors also have a role to play in determining the effect of exposure [9-17].

In cases with CO-induced encephalopathy, tissue hypoxia alone cannot explain the delayed neuropsychiatric manifestations, where immunological and biochemical changes might have a vital role in causing neuronal injury [18,19]. In the animal model experiment, CO poisoning causes immunological cascade activation via biochemical changes of myelin basic protein (MBP) and malondialdehyde, a reactive product of lipid peroxidation. The degradation of MBP occurs in the brain over days and is linked to adaptive immunity activation, which may support delayed neuropsychiatric manifestations [18]. The latent period of neuropsychiatric manifestation is about 2-40 days after the acute phase [20]. In delayed encephalopathy, patients suffer from functional deficiencies, including abnormal personality and cognitive impairment [14]. However, there are no clinical prognostic factors for predicting the development of delayed neuropsychiatric symptoms. Age greater than 36 years, exposure for more than 24 hours, those who did not receive hyperbaric oxygen therapy (HBOT), and patients with cerebellar signs or GCS less than 9 on presentation have all been linked to an increased risk of cognitive impairment [20,21].

The best method for demonstrating the radiological characteristics of CNS involvement is MRI. Common radiological findings include changes in bilateral globus pallidum or widespread cerebral white matter alterations, as well as abnormalities in the basal ganglia and corpus callosum. These changes can occur with or without diffusion restriction field abnormalities [14,22,23]. 

In this case report, the patient developed CO-induced cardiomyopathy on day 2 of the presentation, which was confirmed by echocardiography and cardiac MRI. The imaging was suggestive of regional myocarditis involving the apical segment resembling takotsubo cardiomyopathy. The coronary angiogram was normal. The patient's cardiac status stabilized and completely recovered with full improvement of functional status within four days, which was confirmed by transthoracic echocardiography. In the published literature, the transthoracic echocardiographic changes reported include global left ventricular dysfunction, regional wall hypokinesia, and akinesia, which resembled takotsubo cardiomyopathy [10]. We noted that the patient’s neuropsychiatric manifestation has sub-acutely progressed in one month period from the first presentation, which was compatible with the reported previous study. Furthermore, she had developed typical radiological features similar to previous studies.

## Conclusions

CO poisoning is potentially harmful and may even be lethal. Although the majority of cases have been described as mild, involvement of the cardiovascular and neurological systems contributes to substantial morbidity and mortality. Acute clinical signs of CO poisoning may recover entirely, although neuropsychiatric symptoms may appear later. A high index of clinical suspicion, as well as appropriate radiological imaging, may be of benefit in the early diagnosis of such complications.
